# A commentary on “Development and validation of a clinical decision tool for predicting long-term pain reduction following laparoscopic cholecystectomy in patients with symptomatic cholecystolithiasis; a prospective cohort study”

**DOI:** 10.1097/JS9.0000000000002915

**Published:** 2025-07-07

**Authors:** Ze Xiang, Xingyu Luo, Zhe Yang

**Affiliations:** a Zhejiang University School of Medicine, Hangzhou, China; b Deparment of Hepatobiliary and Pancreatic Surgery, Key Laboratory of Artificial Organs and Computational Medicine in Zhejiang Province, Shulan (Hangzhou) Hospital, Shulan International Medical College, Zhejiang Shuren University, Hangzhou, China; c Department of Hepatobiliary and Pancreatic Surgery, Shulan (Boao) Hospital, Boao, China

*Dear Editor*,

We read with great interest the article by Comes *et al*, which describes the development and external validation of a clinical decision tool to predict long-term pain reduction following laparoscopic cholecystectomy (LC) for symptomatic cholecystolithiasis[1]. This meticulously designed prospective cohort study represents a significant endeavor to address the widely acknowledged challenge of appropriate patient selection for LC. While the authors provide important insights, we believe that several aspects warrant further discussion and future exploration. This study is compliant with the TITAN Guidelines 2025—governing declaration and use of AI[2].

Firstly, the finding that male sex strongly predicts favorable pain outcomes may inadvertently oversimplify sex-related differences. Significant disparities exist in pain perception, health-seeking behaviors, and reporting patterns between males and females[3]. Without considering sex-specific psychosocial factors, hormonal influences, and varying symptom thresholds[4], this model risks perpetuating gender bias rather than offering biologically or clinically meaningful insights. More nuanced, sex-stratified analyses are crucial to clarify whether observed differences reflect true biological variability or sociocultural reporting bias.

Secondly, the model’s dichotomization of several abdominal symptoms (presence/absence of nausea and vomiting, diarrhea, obstipation, heartburn, and post-prandial bloating) likely oversimplifies a complex symptom spectrum. Functional gastrointestinal disorders are not binary. Rather, they exist along a continuum of severity and frequency[5]. Converting these variables into dichotomous ones may sacrifice predictive accuracy and obscure clinically relevant thresholds. Employing continuous or graded symptom scales could enhance model granularity and better reflect the true burden of symptoms in a real-world setting.

Thirdly, although the authors utilized the SECURE trial for external validation, this cohort remained geographically and demographically similar to the development cohort, with both being from Dutch hospitals. Given that healthcare practices, patient demographics, and disease phenotypes vary internationally, the model’s generalizability to non-European or more diverse populations remains uncertain. Transnational external validation is crucial to ensure the model’s robustness across different healthcare settings and ethnic backgrounds[6].

In conclusion, while the proposed model represents a valuable advancement in the personalized selection of LC candidates, caution is needed in its clinical application. Addressing potential gender biases, avoiding dichotomy in symptom assessment, and seeking international validation can jointly enhance its future utility and impact. We commend the authors’ significant contributions and hope that these viewpoints can promote the further improvement of predictive strategies in biliary tract surgeries.

## Data Availability

All data are included in the article.
